# Improved Algorithm for Gradient Vector Flow Based Active Contour Model Using Global and Local Information

**DOI:** 10.1155/2013/479675

**Published:** 2013-09-24

**Authors:** Jianhui Zhao, Bingyu Chen, Mingui Sun, Wenyan Jia, Zhiyong Yuan

**Affiliations:** ^1^School of Computer, Wuhan University, Wuhan, Hubei 430072, China; ^2^Suzhou Institute of Wuhan University, Suzhou, Jiangsu 215123, China; ^3^Department of Neurosurgery, University of Pittsburgh, Pittsburgh, PA 15213, USA

## Abstract

Active contour models are used to extract object boundary from digital image, but there is poor convergence for the targets with deep concavities. We proposed an improved approach based on existing gradient vector flow methods. Main contributions of this paper are a new algorithm to determine the false part of active contour with higher accuracy from the global force of gradient vector flow and a new algorithm to update the external force field together with the local information of magnetostatic force. Our method has a semidynamic external force field, which is adjusted only when the false active contour exists. Thus, active contours have more chances to approximate the complex boundary, while the computational cost is limited effectively. The new algorithm is tested on irregular shapes and then on real images such as MRI and ultrasound medical data. Experimental results illustrate the efficiency of our method, and the computational complexity is also analyzed.

## 1. Introduction

Image segmentation is one of the basic problems of image processing and computer vision, and its purpose is to divide the image into several different areas, while each region has certain characteristics and disjoints with the others. Contour extraction [1–3] is one basic segmenting approach, for which the active contour model (ACM) has been widely studied and applied [4–14]. Existing methods of ACM can be divided into two types [15]: parametric ACMs [4–8, 16, 17] and geometry based ACMs [9–12, 14, 18–20]. Due to effective mathematic methods, parametric ACMs are often faster than geometry based ACMs [11–13]. Geometry based ACMs need higher dimensional functions, which make them slower. Therefore, parametric ACMs are more suitable for certain applications [13, 15], and the novel algorithm proposed in our paper also belongs to the parametric ACMs.

The method of active contour model [4] was proposed by Kass et al. The original ACM has certain disadvantages. First, the external force field of ACM attenuates rapidly when away from image edges, which makes the capturing range of ACM very small; that is, its external force field is very limited. Second, image noise may stop the deformation of active contour where the local minimum energy is obtained and leads the contour to incorrect border; thus, the initial active contour is required to be very near the object boundary. The disadvantages narrow the applications of original ACM, which can only handle the objects without complex boundaries while the initial active contours are not far from the edges.

Based on the original ACM, Xu and Prince proposed the gradient vector flow (GVF) active contour model [7]. The GVF based ACM presents a new external force field, and the diffusing operation makes GVF force field spread to the entire image and thus reflects the static global information. GVF based ACM generates fairly good results even when dealing with image noise and concave edges. The external force field of GVF is a static force field, and it does not change with deformation time or contour's position. External force field of GVF has a large capturing range, and it can drive the active contour to concavities of image edge. However, during the diffusion of external force field, the competing of forces happens. Thus, for the object boundary with very deep concavities, the GVF based ACM still has difficulty to correctly deal with and then obtain good results [15, 21].

To address the problem of converging the active contour to deep concavities of object boundary, the magnetostatic active contour model was proposed by Xie and Mirmehdi [12]. The magnetostatic ACM hypothesizes that electric currents flow through both object boundary and active contour; thus, the active contour is moved toward object boundary under the influence of the generated magnetic field. The magnetostatic ACM is based on static magnetic theory, and it presents a new external force field based on the local information of image. The magnetostatic force can push active contour towards the complicated boundary and thus converge the active contour to very complex object boundary and can also segment multiple objects with only one initial active contour. However, the external force field of magnetostatic method is dynamic; that is, it has to be continuously updated during each time of active contour's deformation, while the calculation of the magnetic force field is relative complex, which makes computation of magnetostatic active contour method slow [13, 22]. Thus, the magnetostatic ACM needs a high computing cost for object segmentation, which limits its applications.

In this paper, an improved GVF based ACM is presented based on the existing methods using both global and local information. The external force field is first computed using the global information of GVF; then, the initial contour is driven to move towards the boundary of object gradually. When the active contour stops, the local information of it is employed to help determine the false part of contour. If the false part exists, the magnetostatic force of it is calculated, and then the external force field is updated to move the active contour for further time. The above steps are repeated until the active contour stops, and there is no false part.

## 2. Preliminary

To illustrate our proposed approach, the methods of ACM, GVF, and magnetostatic ACM are implemented first, and the procedures are described as follows.

### 2.1. Implementation of ACM

For a closed parametric curve *X*(*s*) = [*x*(*s*), *y*(*s*)], *s* ∈ [0,1], ACM defines the energy function:

(1) $$\begin{array}{c} E=\overset{1}{\underset{0}{\int }}\frac{1}{2}\left(\alpha {\left|X^{\prime }\left(s\right)\right|}^{2}+\beta {\left|X^{\prime \prime }\left(s\right)\right|}^{2}\right)+E_{\text{ext}}\left(X\left(s\right)\right)ds, \end{array}$$

where *α* and *β* are the weighting parameters of contour elasticity and rigidity, respectively, *X*′(*s*) and *X*′′(*s*) denote the first and the second derivatives of *X*(*s*) with respect to *s*, and *E*_ext_(*X*(*s*)) is the external energy derived from image and constraint forces so that it has smaller value near the object boundary and bigger value in the other areas.

The minimization of *E* must satisfy the Euler equation:

(2) $$\begin{array}{c} {\alpha X}^{\prime \prime }\left(s\right)-{\beta X}^{\prime \prime \prime \prime }\left(s\right)-\nabla E_{\text{ext}}=0 \end{array}$$

which can be regarded as a force balance equation:

(3) $$\begin{array}{c} F_{\mathrm{int}}+F_{\text{ext}}=0, \end{array}$$

where *F*_int⁡_ = *αx*′′(*s*) − *βx*′′′′(*s*), *F*_ext_ = −∇*E*_ext_, internal force *F*_int⁡_ discourages both stretching and bending, external force *F*_ext_ pulls the active contour to the desired image edges, and finally the curve stops at the position with force balance.

In order to solve (2), the active contour is taken as a function of time *t* and parameter *s*; that is, *X*(*s*, *t*) and the solution of (2) become the solution of:

(4) $$\begin{array}{c} X_{t}\left(s,t\right)=\alpha X^{\prime \prime }\left(s,t\right)-\beta X^{\prime \prime \prime \prime }\left(s,t\right)-\nabla E_{\text{ext}}. \end{array}$$

### 2.2. Implementation of GVF

The GVF based ACM defines a new external force field *F*_ext_^*g*^ = *V*(*x*, *y*), and the new external force field is named gradient vector flow force field. From (4), there is

(5) $$\begin{array}{c} X_{t}\left(s,t\right)=\alpha X^{\prime \prime }\left(s,t\right)-\beta X^{\prime \prime \prime \prime }\left(s,t\right)+V\left(x,y\right). \end{array}$$

An edge map *f*(*x*, *y*) is calculated from the original image *I*(*x*, *y*), and the value of edge map is larger at positions near the image edges. Edge map can be obtained from gray-level images or binary images as

(6) $$\begin{array}{c} f\left(x,y\right)\;=\;-\nabla E_{\text{ext}}^{i}\left(x,y\right), \end{array}$$

where *i* = 1,2, 3, or 4. Edge map has three characteristics: the gradient vector of edge map, that is, ∇*f*, should point to and be perpendicular with the object boundary; the gradient vector of edge map has larger value at object boundaries; in the smooth region of image where little change with the value of *I*(*x*, *y*), ∇*f* is close to 0.

The gradient vector flow force field can be expressed as *V*(*x*, *y*) = (*u*(*x*, *y*), *v*(*x*, *y*)), and its energy function is

(7) $$\begin{array}{c} \varepsilon =\iint \mu \left(u_{x}^{2}+u_{y}^{2}+v_{x}^{2}+v_{y}^{2}\right)+{\left|\nabla f\right|}^{2}{\left|V-\nabla f\right|}^{2}dx\;dy. \end{array}$$

The energy function follows a standard principle that, in the absence of gradient vector field, the smoothness of active contour is ensured. When value of ∇*f* is small, the energy function is determined by the sum of the squares of partial derivatives of gradient vector force field. When value of ∇*f* is large, the energy function is determined by |∇*f*|^2^|*V*−∇*f*|^2^ and is minimized by *V* = ∇*f*. Therefore, *V* nearly equals to the gradient of edge map where gradient value is large and changes little where gradient value is small. As the weighting parameter, *μ* is set according to the proportion of noise in image, that is, more noise with larger value of *μ*.

### 2.3. Implementation of Magnetostatic ACM

The magnetostatic ACM is based on level-set approach, while the evolution equation of level-set function *ϕ* is

(8) $$\begin{array}{c} \frac{\partial \phi }{\partial t}+F\left|\nabla \phi \right|=0, \end{array}$$

where *F* is the speed function with certain form.

The active contour of magnetostatic based ACM can be represented implicitly as

(9) $$\begin{array}{c} C=\left\{x\in \Omega \;|\;\phi \left(x,t\right)=0\right\}, \end{array}$$

where *C* is the active contour, *Ω* is the image domain, and *ϕ*(*x*, *t*) is the level-set function at time *t*.

As for its applying in image segmentation, deformation of the active contour of magnetostatic ACM is associated with the following partial differential equation:

(10) $$\begin{array}{c} \frac{\partial \phi }{\partial t}=-\left|\nabla \phi \right|\left(as\left(x\right)\nabla \left(\frac{\nabla \phi }{\left|\nabla \phi \right|}\right)+\left(1-a\right)f\left(x\right)\right), \end{array}$$

where *a* is weighting parameter, *s*(*x*) is stop function, and *f*(*x*) is the external force field of magnetostatic ACM.

## 3. Improved GVF Active Contour Model

Then, the new improved algorithm for GVF based ACM is implemented, during which both false contour determination and updating of external force field are difficult problems.

### 3.1. Basic Idea

To make the active contour converge to deep concavities of object boundary and spend less time during deformation, an improved GVF based ACM is proposed. Our approach combines advantages of GVF based ACM and magnetostatic ACM. Global information is considered with GVF based ACM to generate the external force field, which drives the active contour to object boundary. When there is false part of the stopped contour, local information around the false part is considered with the help of magnetostatic ACM to compute the magnetostatic force and add to the existing external force field; then the external force field is updated.

Therefore, our method has a semidynamic external force field, which is dynamic when there is false part of the active contour, and magnetostatic force is added and is static when the active contour is moved. Our method uses both global and local information of image; that is, the global information is used when GVF external force field is computed, while the local information is used when false part of active contour is judged and the magnetostatic force is computed.

### 3.2. False Contour Determination

**Figure 1:**
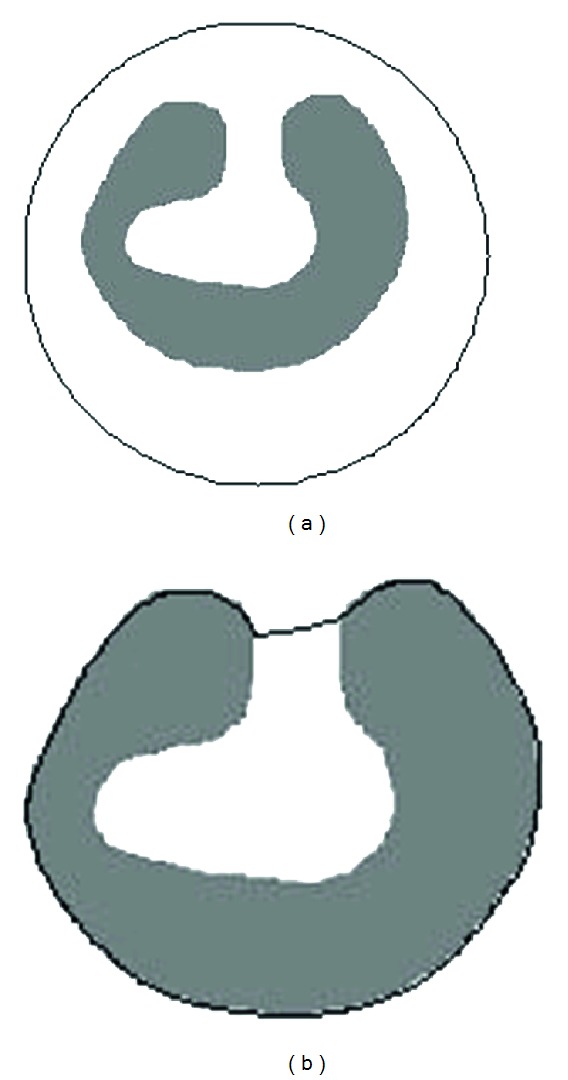
Extracted boundary with deep concavity using GVF based ACM. (a) Object and initial active contour. (b) Result after 150 iterations.

GVF based ACM can converge the active contour to edges of complex object, but it cannot deal with deep concavities of boundary.  Figure 1 shows the extracted boundary of an object with deep concavity using GVF; that is, Figure 1(a) is the object and the initial active contour, while Figure 1(b) is the result after 150 times of contour movement. From the result, it can be found that part of the active contour stops at the entrance of deep concavity where the minimum energy is met, and such part is the false active contour. Only if the false part is determined and the local external force field is updated, the false contour can be further moved into the concavity.

**Figure 2:**
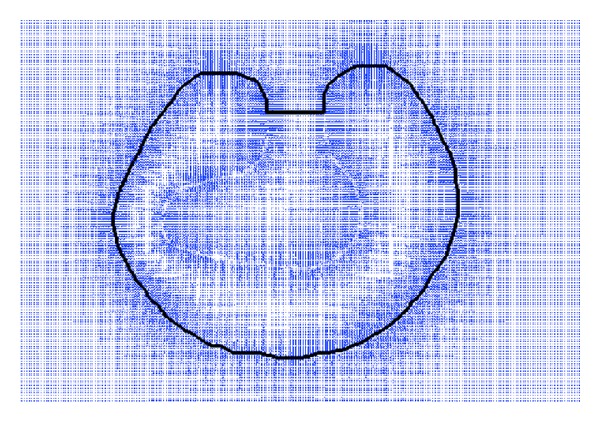
External force field of GVF and stopped active contour.

**Figure 3:**
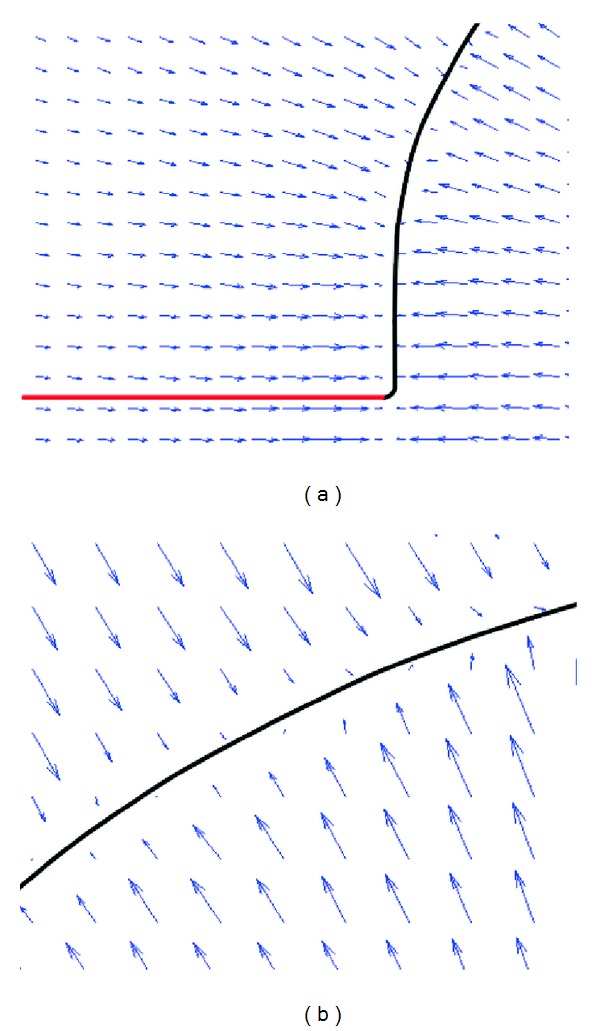
The locally enlarged regions of external force field.

Figure 2 is the external force field of GVF and the stopped active contour. To illustrate the force field clearly, the local regions of external force field are enlarged and are shown in Figure 3. The local region around right side of the entrance to the deep concavity is shown in the left image of Figure 3. We can find that the black part of contour has moved to the object boundary, and it is real active contour, while the red part has stopped at the entrance, and it is false active contour. Also, it can be found that most angles between the real active contour and the force vectors of the external force field are near 90 degrees, while most angles between false active contour and vectors of external force field are near 0 degree.

In [22], the false active contour is judged when one angle between active contour and external force field is less than a predefined threshold value. But sometimes this method can make mistakes, since there are certain irregular directions of external forces around the real active contour. As shown in the right image of Figure 3, the angles between irregular external forces and real active contour are also very small. To avoid the inaccurate judgements, a more precise method is needed to determine the false part of active contour.

In our new judgement method for false part, the angles between the tangent vector of each point in active contour and the 9 external forces around it, in the local 3 × 3 area, are calculated. If every angle is less than a threshold value, the point in contour is judged as false active contour point. If the amount of false contour points in a part of active contour is more than a threshold value, it is judged as the false part of active contour. The new false active contour determination method makes the judgment more accurate, even in the cases of irregular directions of external forces.

Judgement formula of the false active contour point is

(11) $$\begin{array}{c} FP_{X(s)}=\{\begin{array}{cc} 1 & \text{if}\;\;T_{s,v(x,y)}\leq \theta ,\\ 0 & \text{if}\;\;T_{s,v(x,y)\;}>\theta , \end{array} \end{array}$$

where *s* ∈ [0,1], *T*_*s*,*v*(*x*,*y*)_ is the maximum angle between active contour *X*(*s*) (tangent vector of the point under processing in active contour) and the around external forces *v*(*x*, *y*) in the local 3 × 3 region, and *θ* is the predefined angle threshold value. Consider that *FP*_*X*(*s*)_ = 1 means that the point is false active contour point, while *FP*_*X*(*s*)_ = 0 means that this point is not a false active contour point. *T*_*s*,*v*(*x*,*y*)_ can be represented as

(12) $$\begin{array}{c} T_{s,v(x,y)}=\max\left(t_{s,v(i,j)}\right), \end{array}$$

where *i* = *x* − 1, *x*, *x* + 1, while *j* = *y* − 1, *y*, *y* + 1.

Formula (11) is used to scan the whole active contour, and all false active points are recorded. Thus, the active contour is divided into *l* segments; that is, there are *l* dividing points *p*_*i*_ where *i* ∈ [1, *l*], and the related *l* segments *L*_1_ = (*p*_1_, *p*_2_), *L*_2_ = (*p*_2_, *p*_3_),…, *L*_*l*_ = (*p*_*l*_, *p*_1_). Then, amount of false active contour points is calculated for each segment. If the amount of false active contour points is greater than a predefined threshold, the corresponding segment is a false part of active contour. The judgement formula is

(13) $$\begin{array}{c} {FC}_{i}=\{\begin{array}{cc} 1 & \text{if}\left(\overset{}{\underset{X(s)\in L_{i}}{\sum }}FP_{X(s)}\right)*\frac{l}{U}>\eta ,\\ 0 & \text{if}\left(\overset{}{\underset{X(s)\in L_{i}}{\sum }}FP_{X(s)}\right)*\frac{l}{U}\leq \eta , \end{array} \end{array}$$

where *U* is the length of active contour and *η* is threshold value for the amount of false active contour points of one segment. If *FC*_*i*_ = 1, *L*_*i*_ is a false part of active contour; and if *FC*_*i*_ = 0, *L*_*i*_ is not a false part.

**Figure 4:**
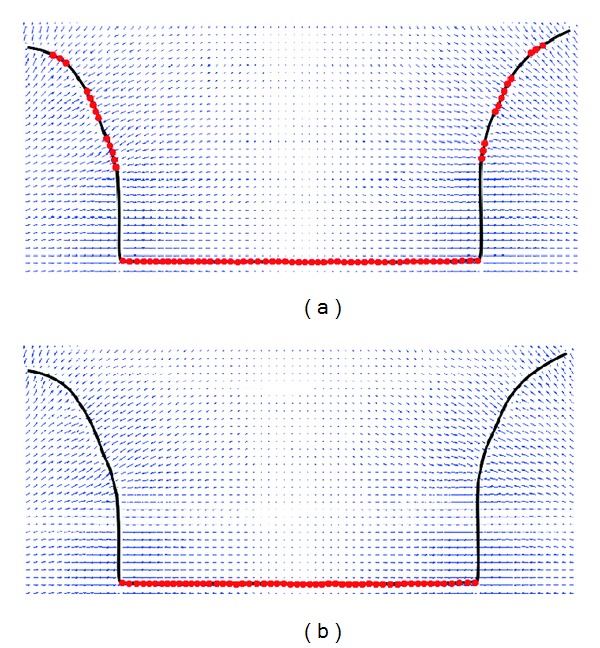
Judgements of false parts in active contour by [22] and our new method.

Judgements of false parts in active contour are displayed in Figure 4. The top image is the determined false part (in red color) of active contour from the method of [22], while the bottom image shows the determined result from our new method. From the comparison, it can be found that our new method can detect the false part of active contour with higher accuracy, and only the false part needs further processing to approach towards the deep concavity.

### 3.3. Updating of External Force Field

In our improved GVF active contour model, the external force field is made of two parts: one is the static GVF force field, the other is the dynamic magnetic force field. The basic external force field *V*(*x*, *y*) of GVF based ACM is calculated by minimizing the energy function (7); then the initial active contour deforms under the influence of the force field. When the false part appears in the deformed contour, the magnetic force field *F*(*x*, *y*) is computed; then the new external force field is obtained through combination of the basic external force field and the magnetic force field. And then the active contour deforms for the next iteration under the influence of the new external force field.

The calculation of the magnetic force field is based on the magnetostatic theory. Assume that there are electric currents in both the object boundary and the false active contour. The magnetic field produced by false active contour is ignored, and only the magnetic field generated from object boundary is considered. In the magnetic field from object boundary, the false active contour is affected by the produced magnetic force and moved towards the object boundary. The electric current of object boundary is obtained through rotating the gradient of image for 90 degrees using the following equation:

(14) $$\begin{array}{c} O\left(x,y\right)={\left(-1\right)}^{\lambda }\left(-I_{y}\left(x,y\right),I_{x}\left(x,y\right)\right), \end{array}$$

where *I*_*x*_(*x*, *y*) and *I*_*y*_(*x*, *y*) are partial derivatives of image *I*(*x*, *y*) in *x* and *y*. When *λ* = 1, direction of the electric current is counter clockwise; when *λ* = 2, direction of the electric current is clockwise. 

The magnetic field produced by electric current of object boundary is

(15) $$\begin{array}{c} B\left(x,y\right)=\frac{\mu_{0}}{4\pi }\underset{c\in f(c)}{\sum }I_{f(c)}\Gamma \left(c\right)*\frac{{\hat{R}}_{(x,y),c}}{R_{(x,y),c}^{2}}, \end{array}$$

where *c* is a point in object boundary, *f*(*c*) is the object boundary, Γ(*c*) is the electric current vector at point *c*, and ${\hat{R}}_{(x,y),c}$ is the unit vector from (*x*, *y*) to *c*, while *R*_(*x*,*y*),*c*_^2^ is the distance between (*x*, *y*) and *c*. Suppose that the electric current of false active contour is *I*_*s*_; the magnetic force generated from magnetic field of object boundary is

(16) $$\begin{array}{c} F\left(x,y\right)=I_{s}\Upsilon \left(s\right)*B\left(x,y\right), \end{array}$$

where *Υ*(*s*) is the direction of electric current of false active contour. The magnetic force *F*(*x*, *y*) exerted on the false active contour is always vertical to it while points towards inside or outside of the active contour.

After computation of the magnetic force field around the false active contour, the basic external force field *V*(*x*, *y*) is added with the magnetic force field *F*(*x*, *y*); then the new external force field *V*_new_(*x*, *y*) is obtained as

(17) $$\begin{array}{c} V_{\text{new}}\left(x,y\right)=V\left(x,y\right)+kF\left(x,y\right), \end{array}$$

where *k* is the weighting parameter of *F*(*x*, *y*), and it is used to adjust the effect from the magnetic force.

**Figure 5:**
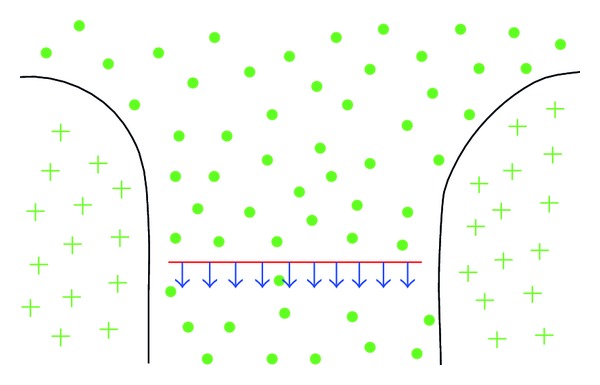
Magnetic force field of the false active contour.

Figure 5 displays the force field around false active contour produced from the electric current of object boundary. The black curve represents object boundary, the green + denotes magnetic field from object boundary going perpendicularly into the image plane, and the green • denotes the magnetic field coming perpendicularly out of image plane, while the blue arrows represent the magnetic forces with vertical directions to the false active contour, which is denoted with a red line.

**Figure 6:**
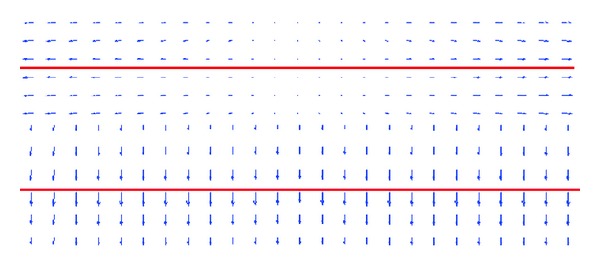
External force field at the entrance of deep concavity.

As shown in the top image of Figure 6, there is no downward component of basic external force field *V*(*x*, *y*) at entrance of deep concavity where the false active contour stops. As shown in the bottom image of Figure 6, after adding the magnetic force field, now there is downward component of updated external force field *V*_new_  (*x*, *y*), which helps converge the false active contour into deep concavity.

## 4. Experimental Results

To test the performance of our new method, experiments are performed including setting of parameters, comparisons with existing ACMs, and testing on real images.

### 4.1. Setting of Parameters

The initial active contour is generated by interpolation of a series of manually marked points for object. In our improved GVF active contour model, the parameters need to be set are *α*, *β*, *μ*, *λ*, *k*, *θ*, *l*, and *η*.

The elastic parameter *α* and rigid parameter *β* are used to control integrity and smoothness of the active contour, that is, ensure that the active contour is a complete closed curve with good smoothness during the deformations. In our work, we set *α* = 0.6 and *β* = 0.3. Parameter *μ* is the smoothing parameter, and it is set according to image noise. Generally, *μ* < 0.25, and we set *μ* = 0.2. Parameter *λ* controls the direction of electric current in object boundary, and when *λ* = 1, the electric current is counter clockwise; when *λ* = 2, the electric current is clockwise. If the initial active contour is inside or outside of the object, the directions of electric currents of object boundary and active contour must be the same. If the initial active contour intersects with the object boundary, the electric current directions of object boundary and active contour must be different. Only in this way, the active contour can be moved to object boundary; otherwise it is moved away from object boundary. Parameter *k* is used when adding the basic external force field and the magnetic force field. It adjusts the weight of magnetic force field in the updated external force field, and we set *k* = 0.6. Angle *θ* is the threshold value to judge false active contour points. Too large value of *θ* can take the real active contour points as false ones, while too small value of *θ* can take false active contour points as real ones; thus, we set *θ* = 20. Parameter *l* is the number of segments used when judging the false active contour, and it is set based on the whole length of the active contour. Since too long or too short segment will affect the judgment accuracy of false active contour, *l* is set to make each segment have 50 active contour points. Percentage *η* is the threshold value for false active contour determining. For each active contour segment, if the proportion of false active contour points is more than *η*, the segment is taken as false active contour segment. In our work, we set *η* = 0.8.

### 4.2. Comparison with Existing ACMs

**Figure 7:**
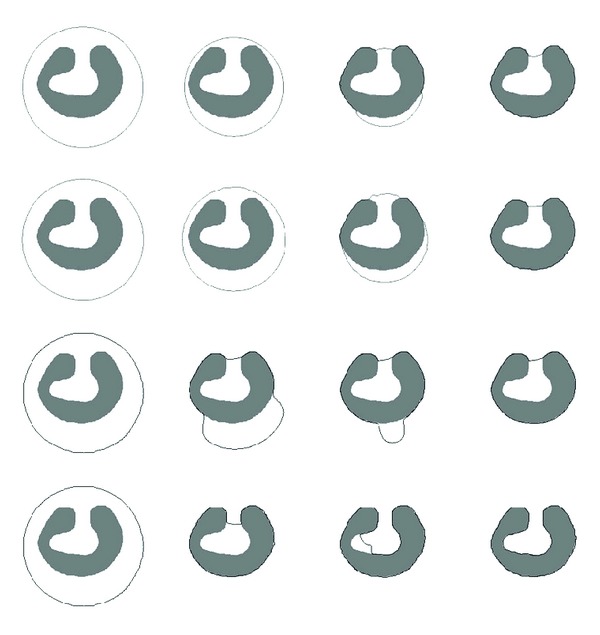
Results on object with one deep concavity.

**Figure 8:**
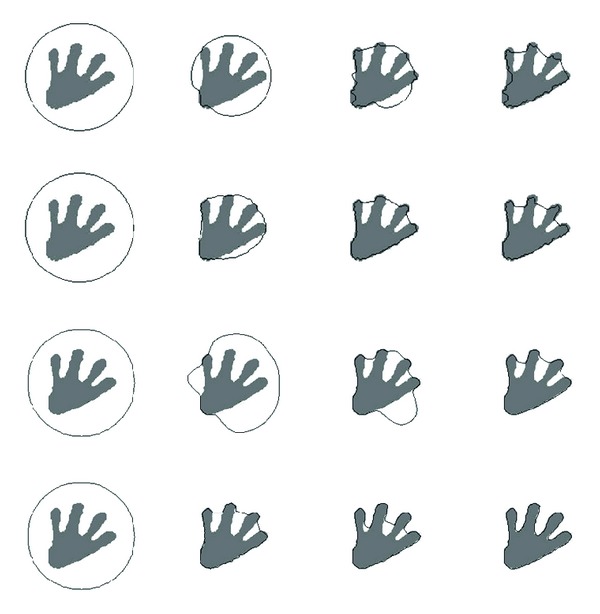
Results on object with multiple deep concavities.

Existing ACMs and our method are tested on object with one deep concavity, and the results are shown in Figure 7. The 1st row results from balloon active contour model, the 2nd row results from distance vector flow active contour model, the 3rd row results from gradient vector flow active contour model, and the 4th row results from our method. The 1st column is the object and the initial active contour, the 2nd column is the results after 50 (150 of our method) iterations, and the 3rd column is the results after 100 (250 of our method) iterations, while the 4th column is the results after 150 (350 of our method) iterations. Then, the methods are tested on the object with multiple deep concavities, and related results are shown in Figure 8 with the same arrangements as Figure 7. From the results, it can be found that our method has the ability to converge the active contour to complex object border, even for the object boundary with very deep concavities.

Compared with GVF based ACM, when the initial active contour stops deformation, our method continues to scan the active contour and search for the false part. Then, magnetic force field around the false part is computed, and the new external force field is generated. Thus, the active contour has opportunities to be adjusted for more times until there is no false part. Therefore, computing cost of our method is higher than these existing ACMs. However, our method performs faster than the magnetostatic ACM. The magnetostatic ACM calculates magnetic force of every point in active contour and updates the external force field for each deformation, while our method calculates the magnetic force field only when the false active contour appears. Our method updates the external force field only if needed, which makes its computational efficiency better than that of magnetostatic ACM.

### 4.3. Results on Real Images

**Figure 9:**
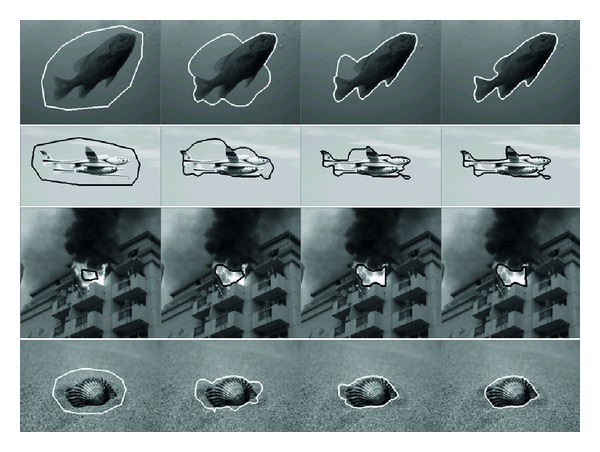
Experiments on real images with complex objects.

**Figure 10:**
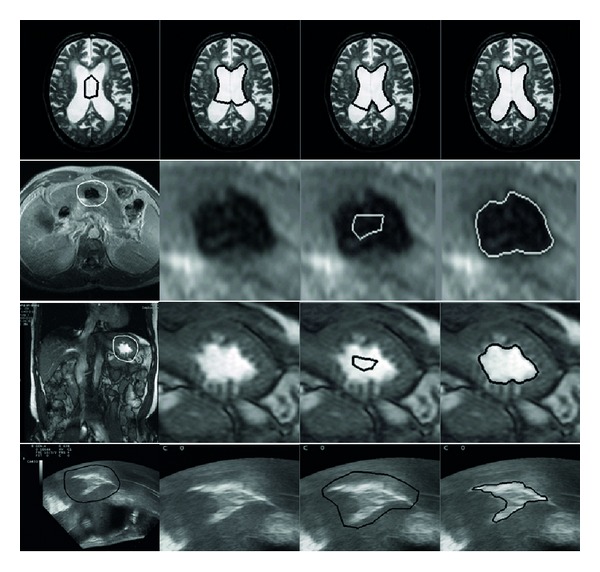
Experiments on MRI and ultrasound images.

Our improved GVF based ACM are tested on some real images of objects with complex boundaries, for example, fish, plane, fire, shell, as shown in Figure 9, and the initial active contours successfully converge to the deep concavities after a number of iterations. Then, our method is used to process medical data including MRI and ultrasound images, as illustrated in Figure 10. The active contours move to image edges correctly, and the object boundaries are extracted with high precision. From the 2nd to 4th rows, the 1st and 2nd columns are regions of interest and the 3rd column are initial active contours, while the 4th column are correctly converged active contours.

## 5. Conclusion

GVF based ACM has the large capture range due to its external force field, but it has difficulty to converge the active contour to deep concavities of object boundary. In this paper, we proposed an improved method for GVF with the help of magnetic force field from magnetostatic ACM. Our method uses global information to calculate the basic GVF external force field to adjust the initial active contour and then uses local information to determine the false part of active contour and calculate the magnetic force field for the false contour. The new external force field is obtained through addition of GVF external force field and magnetic force field and is used to drive the active contour to converge again. These operations are repeated until there is no false part of the stopped active contour. The key techniques of our method include a new algorithm to determine the false part of active contour and a new algorithm to update the external force field.

Based on a lot of experiments, the new method has been tested and analyzed. Compared with the existing ACMs, our method has better performance for edge extraction, even for boundaries with very deep concavities. Our method also has higher computational efficiency than magnetostatic ACM.
